# Sino-Austrian High-Tech Acupuncture Network—Annual Report 2014

**DOI:** 10.3390/medicines2010001

**Published:** 2014-12-24

**Authors:** Gerhard Litscher

**Affiliations:** Research Unit for Complementary and Integrative Laser Medicine, Research Unit of Biomedical Engineering in Anesthesia and Intensive Care Medicine, and TCM Research Center Graz, Medical University of Graz, Auenbruggerplatz 29, 8036 Graz, Austria; E-Mail: gerhard.litscher@medunigraz.at; Tel.: +43-316-385-13907; Fax: +43-316-385-13908.

**Keywords:** high-tech acupuncture, network, annual report, China, Austria

## Abstract

The Sino-Austrian High-Tech Acupuncture Research Network was founded in 2005 and has been growing ever since. The network comprises many partners from China and is highly involved in research and publication activities. This report introduces the network’s activities in the year 2014.

Within the last year, the Sino-Austrian High-Tech Acupuncture Network (Figure 1) has grown almost exponentially. The network, which was initiated and founded by Prof. DDr. Gerhard Litscher from the Medical University of Graz, Austria, in 2005, comprises many partners from China.

**Figure 1:**
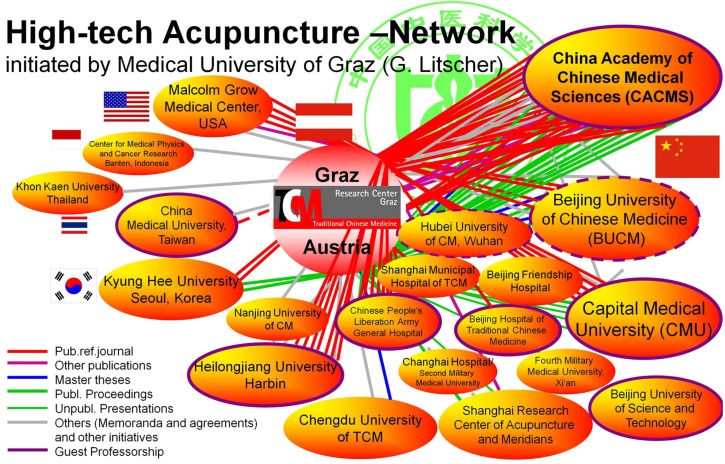
The Sino-Austrian high-tech acupuncture research network with all its members.

Apart from the main partner, the China Academy of Chinese Medical Sciences in Beijing, there were important cooperation with the Capital Medical University in Beijing and also with the Heilongjiang University of Chinese Medicine in Harbin [1,2] during the last year. In the following, some milestones from the year 2014 are listed chronologically:

**1 January 2014**: The Sino-Austrian Research Center for High-Tech Acupuncture and Clinical & Experimental Integrative Medicine is established at the Beijing Hospital of Traditional Chinese Medicine affiliated with the Capital Medical University. The center is headed by Prof. Gerhard Litscher (director of the Center, Austria) and Prof. Wang Lin-Peng (director, China) (Figure 2). The two vice-directors are Priv.-Doz. Lu Wang (Austria) and Prof. Liu Cun-Zhi (China). One of this year’s joint projects deals with the investigation of acupuncture effects on autonomic balance in adult tinnitus patients. This research project is registered as a Controlled Trial, and the design had been pre-published. In conclusion, the research teams found that needle acupuncture with so-called deqi may have better effects on tinnitus patients than acupuncture without deqi sensation.

**Figure 2:**
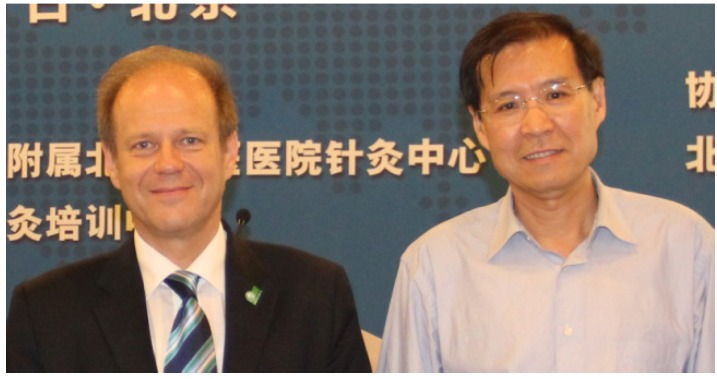
The heads of the Sino-Austrian Research Center for High-Tech Acupuncture and Clinical & Experimental Integrative Medicine. L: Prof. Gerhard Litscher, Austria; R: Prof. Wang Lin-Peng, China.

**14 May 2014**: The Nanjing University of Chinese Medicine hosts researchers from the TCM Research Center at the Medical University of Graz. A discussion about the integration of this famous TCM University in the acupuncture network takes place in a very friendly atmosphere (Figure 3).

**Figure 3:**
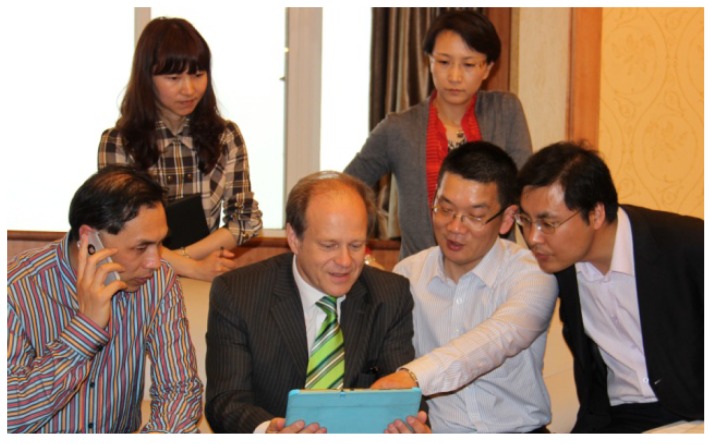
Cooperation talks at Nanjing University of Chinese Medicine.

**16–18 May 2014**: The 1st Annual World Congress of High-Tech Acupuncture and Integrative Medicine is organized in Nanjing. High-tech acupuncture is a very successful example of a cross-over approach between tradition and innovation. This annual World Congress shall serve as an opportunity to present and discuss the state of the art of multidisciplinary approaches to modernization of integrative medicine, especially traditional Chinese medicine and acupuncture. This World Congress was held together with BIT’s 7th World Cancer Congress (WCC-2014) and BIT’s 5th Annual World Congress of NeuroTalk-2014. These three conferences brought together more than 400 participants from 36 countries (Figure 4a). Within the keynote forum, lectures were given by the organizer and initiator (Prof. G. Litscher, President of ISLA (Science & Research), Austria; Prof. Dr. Xiaomin Wang, Pro-Vice-Chancellor, Capital Medical University, China (Figure 4d); Prof. Dr. Ying Xia, Vice-Chairman at The University of Texas Medical School at Houston, USA; and Dr. M. Weber, President of ISLA (Medical and Clinical Applications), Germany. The lecture of Gerhard Litscher focused on the latest innovative aspects that underline the further enhancement and development of high-tech acupuncture. Special emphasis was given to acupuncture innovations, e.g. teleacupuncture, laser acupuncture, and laser therapy in animal experimental and human clinical studies. Recent investigations concerning bioengineering assessment and modernization of acupuncture based on innovative technology were also presented. President Xiaomin Wang presented animal and human experimental research using electroacupuncture (EA) in the treatment of Parkinson’s disease (PD). He found that high-frequency EA stimulation with 100 Hz improved motor impairments by decreasing corticostriatal glutamatergic transmission in 6-OHDA-lesioned rats. He also presented results of PD patients treated with EA. Prof. Ying Xia referred to his research concerning acupuncture and stroke, which is one of the leading causes for neurological disability and death in the world. He introduced some clinical and basic studies, which suggest that acupuncture might provide a new opportunity for stroke treatment. Dr. Michael Weber presented the latest developments in integrative laser medicine and therapy. Honored leaders and distinguished guests from all over the world came to attend the congress. Based on the warm support and suggestions from the participants, the organizers (BIT and ISLA) are confident that HTA&IM-2015 will be even better and more successful than HTA&IM-2014.

**Figure 4:**
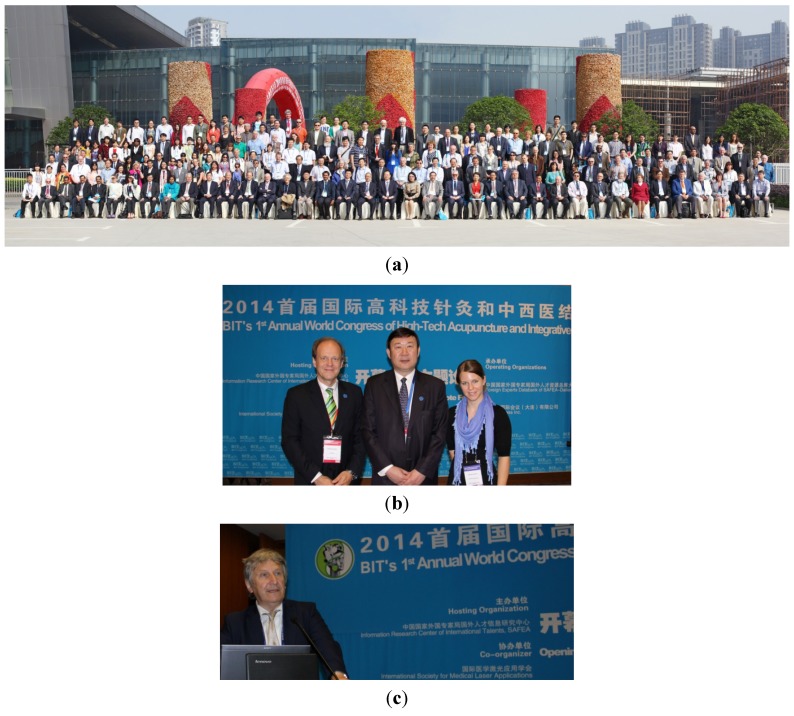

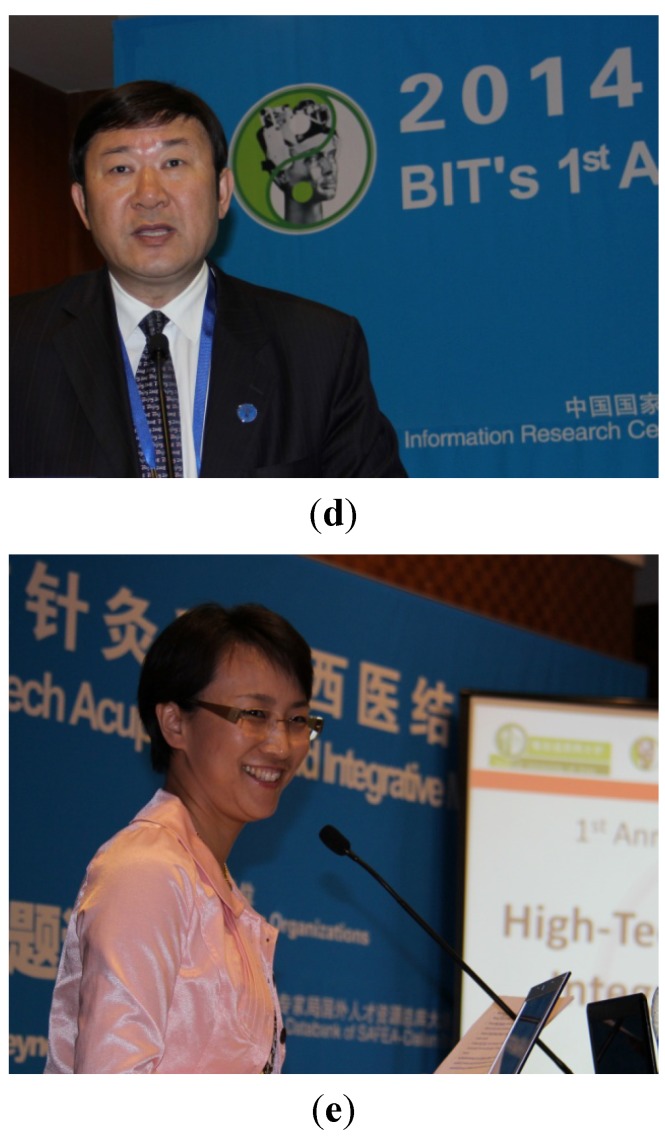
(**a**) Group photo of the High-Tech Acupuncture and Integrative Medicine congress in Nanjing, along with participants of the other congresses. (**b–e**) Impressions from the HTA&IM-congress 2014.

HTA&IM-2015 will take place in Hangzhou, China, from 22–24 May. Several renowned top-experts already agreed to participate and we are sure to establish this conference as an annual meeting point for researchers and practitioners. This is also a cordial invitation to the readers of this report to become an active part of this event. Further information can be found at http://www.bitlifesciences.com/HTA&IM2015 and http://litscher.info.

**12–13 June 2014**: High-tech acupuncture is also one of the topics at the 2014 Beijing International Acupuncture and Neurology Seminar (Figure 5), hosted by Prof. Wang Lin-Peng from Capital Medical University.

**Figure 5:**
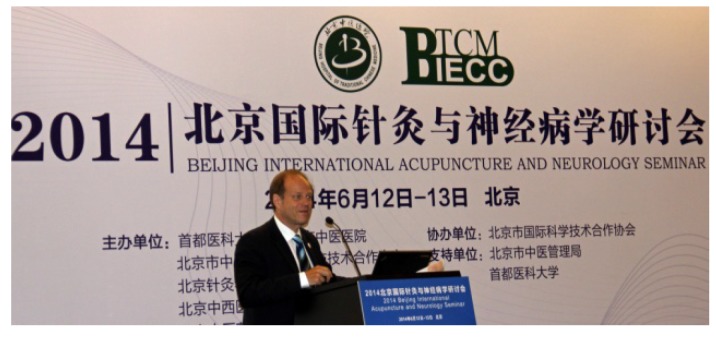
Prof. Gerhard Litscher speaking at the Beijing International Acupuncture and Neurology Seminar.

**13 June 2014**: At the China Academy of Chinese Medical Sciences, a meeting of five Eurasia-Pacific scholarship awardees takes place (Figure 6). All these scholarship holders had visited the TCM Research Center Graz at Medical University of Graz for several months over the last few years.

**Figure 6:**
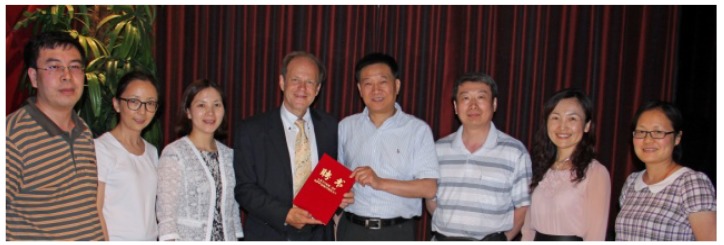
Former and current Eurasia Pacific Uninet scholarship awardees.

**16 June 2014**: Lectures are held by the project leader, not only in Mainland China, but also in Taichung and Taipei (Figure 7) [3,4].

**Figure 7:**
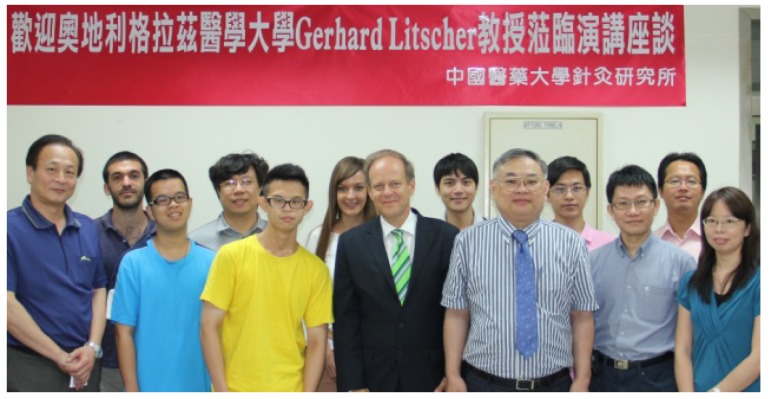
Prof. Litscher with some of his interested audience.

**18 June 2014**: A close collaboration is born in Wuhan. President Prof. Dr. Wang Hua (Figure 8a) and the Chinese project leader Prof. Dr. Liang Fengxia with her team (Figure 8b) intend to have joint research activities within the field of heart rate variability and clinical studies. The next meeting is already planned for January 2015. Prof. Litscher will be awarded as Visiting Professor in Wuhan.

**Figure 8:**
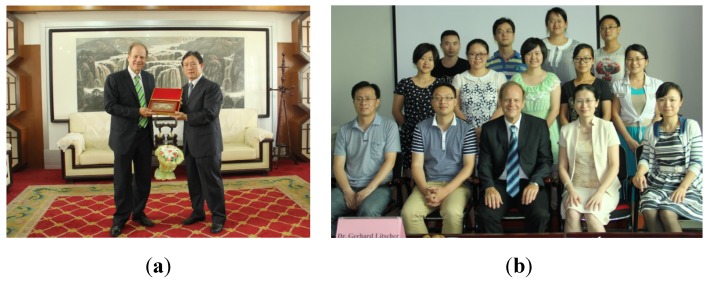
(**a**) Meeting of the initiator of the High-Tech Acupuncture Research Network and the President of Hubei University of Chinese Medicine, Prof. Wang Hua. (**b**) Prof. Liang Fengxia and members of her research team.

**20 June 2014**: The long-lasting cooperation with the chairman of the Military Acupuncture Training Center, Prof. Shi Xian (Figure 9), continues. This year, several studies concerning basic mechanisms of acupuncture and also clinical studies on this topic were published [5,6].

**Figure 9:**
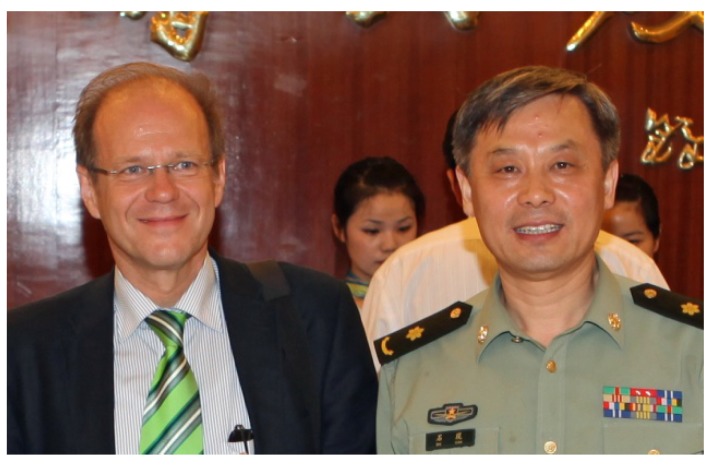
The chairman of the Military Acupuncture Training Center, Prof. Shi Xian.

**25–26 August 2014**: At the 2nd International Conference and Exhibition on Traditional & Alternative Medicine in Beijing (Figure 10) studies in cooperation with the Military Acupuncture Training Center (see above) are presented.

**Figure 10:**
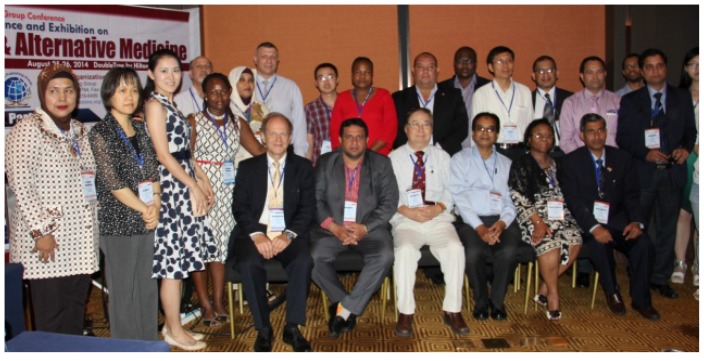
Speakers and participants of the 2nd International Conference and Exhibition on Traditional & Alternative Medicine in Beijing.

**3–5 October 2014**: Austrian members of the TCM Research Center Graz at the Medical University of Graz (Figure 11a) and a member of the high-tech acupuncture network from the China Academy of Chinese Medical Sciences in Beijing (Figure 11b) present their research results at the 8th International Workshop on TCM of the German-Chinese Research Foundation for Traditional Chinese Medicine (DCFG-TCM). Prof. Litscher from Austria is elected German Vice-President of this society.

**Figure 11:**
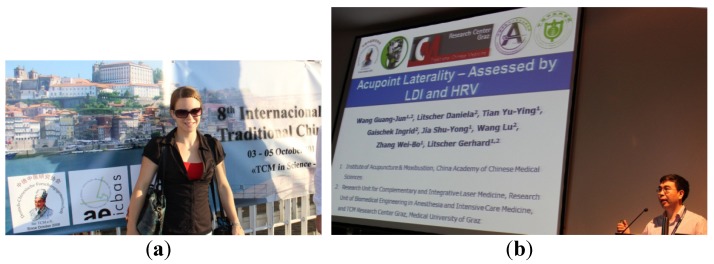
(**a**) and (**b**) Members of the Sino-Austrian High-Tech Acupuncture Research Network at the 8th International Workshop on TCM of the German-Chinese Research Foundation for Traditional Chinese Medicine in Porto, Portugal.

**21 October 2014**: Four members of the high-tech acupuncture network are part of a delegation attending the 1st Sino-Austrian-Croatian TCM Meeting at the University of Zagreb, Croatia (Figure 12). Future aspects of collaboration are discussed and planned. We would like to thank Eurasia Pacific Uninet for the support of this scientific activity.

**Figure 12:**
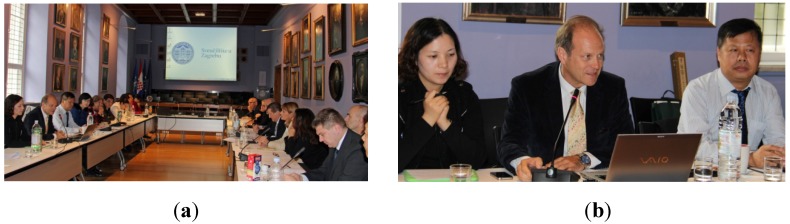
(**a**) and (**b**) Cooperation talks in Zagreb, Croatia.

**25 October 2014**: In Vienna, the 2014 meeting of the Sino-Austrian TCM Joint Research Projects is organized by Eurasia Pacific Uninet (Figure 13). Details concerning the collaboration on the topic “Prevention and early intervention of chronic diseases with TCM”, and especially high-tech acupuncture, are discussed.

**Figure 13:**
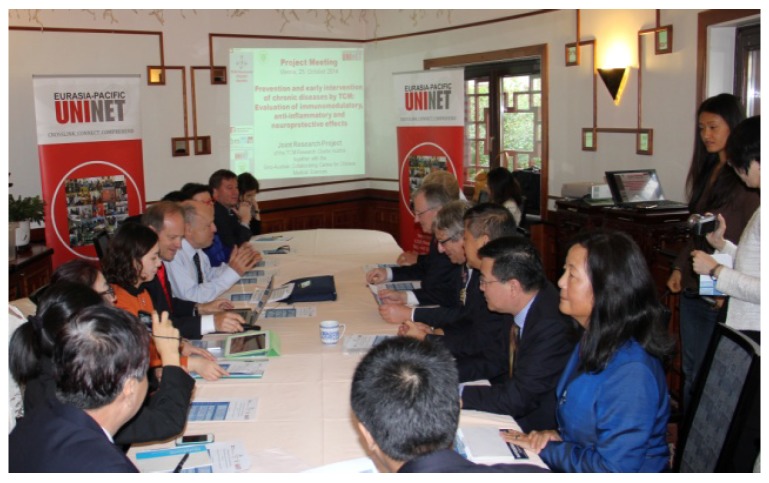
Meeting of the Austrian and some of the Chinese project leaders of the joint Sino-Austrian TCM research projects.

**October–December, 2014**: Assoc. Prof. Guangjun Wang, MD PhD, from the China Academy of Chinese Medical Sciences (Figure 14a), visits the TCM Research Center Graz with a Eurasia Pacific Uninet post-doc scholarship. He also participates in modern laser research (Figure 14b) at the Medical University of Graz [7,8,9].

**Figure 14:**
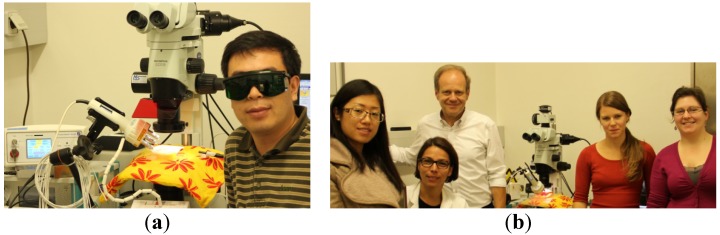
(**a**) This year’s Eurasia Pacific Uninet scholarship holder, Prof. Wang Guangjun; (**b**) Laser measurements in cooperation with the Institute of Pathophysiology and Immunology at the Medical University of Graz.

The Sino-Austrian High-Tech Acupuncture Research Network at the Medical University of Graz is open for further members, and it should be mentioned clearly that the important research activities (54 SCI listed publications within 2012–2014, among them three editorials [10,11,12]) would not have been possible without the support of Eurasia Pacific Uninet.
